# Clinical response of acute idiopathic polyradiculoneuritis treated with therapeutic plasma exchange in four dogs

**DOI:** 10.1093/jvimsj/aalag090

**Published:** 2026-05-18

**Authors:** Valentina E R Dazio, Claudia Iannucci, Franziska B Meyer, Lorenzo Golini, Alessio Vigani

**Affiliations:** Department of Small Animals, Division of Emergency and Critical Care Medicine, Vetsuisse Faculty, University of Zurich, Zurich 8057, Switzerland; Department of Small Animals, Division of Emergency and Critical Care Medicine, Vetsuisse Faculty, University of Zurich, Zurich 8057, Switzerland; Department of Small Animals, Division of Emergency and Critical Care Medicine, Vetsuisse Faculty, University of Zurich, Zurich 8057, Switzerland; Department of Neurology, Vetsuisse Faculty, University of Zurich, Zurich 8057, Switzerland; Department of Small Animals, Division of Emergency and Critical Care Medicine, Vetsuisse Faculty, University of Zurich, Zurich 8057, Switzerland

**Keywords:** acute idiopathic polyradiculoneuritis, Guillain–Barré syndrome, immune-mediated polyneuropathy, lower motor neuron disease, membrane-based plasmapheresis

## Abstract

Acute idiopathic polyradiculoneuritis (AIP) is an immune-mediated peripheral neuropathy in dogs for which no specific treatment has been established in veterinary medicine. In human medicine, therapeutic plasma exchange (TPE) is a standard treatment for Guillain–Barré syndrome, a clinically analogous condition. In this case series, we describe 4 dogs diagnosed with AIP and treated with TPE. All dogs received 3 consecutive TPE sessions, processing between 4.2 and 4.8 total dog plasma volumes. Motor function improvement was observed after the first session in all cases, with near-complete neurological recovery at discharge. No adverse effects were reported. During follow-up (2-12 months), 3 dogs remained disease-free, and 1 dog experienced a recurrence that subsequently resolved. These cases suggest that TPE might promote rapid clinical improvement in dogs with AIP, representing a promising emergency treatment option for dogs with severe and rapidly progressive neurological deficits.

## Introduction

Acute polyradiculoneuritis is a neurological disease associated with peripheral neuropathy in dogs.^1–4^ The pathophysiology of the disease appears to be immune-mediated in nature, with possible triggering factors such as vaccination, raccoon bites, or infectious diseases (eg, *Toxoplasma* spp. or *Campylobacter jejuni*).^2^^,^^4–7^ The most commonly described form in dogs is idiopathic, with no identifiable trigger or known genetic predisposition.^1^^,^^4^

Acute idiopathic polyradiculoneuritis (AIP) is characterized by an acute onset and rapidly progressive course, with clinical signs ranging from generalized weakness to tetraparesis, dysphonia, and, in severe cases, respiratory muscle paralysis.^2^^,^^4^^,^^7–9^

Diagnosis is based on clinical history, neurological examination, electrophysiology, and the exclusion of other causes of peripheral neuropathy.^2^^,^^8^ The clinical course of AIP usually extends over several weeks to months.^8^

There is currently no scientifically validated consensus on the optimal therapeutic management of AIP. Standard treatment focuses on supportive care, including physiotherapy and close monitoring of neurological function.^7^^,^^8^ The use of immunosuppressive corticosteroids has been described, but their impact on clinical outcome remains controversial.^1^^,^^7^ Intravenous administration of immunoglobulin (IVIg) is anecdotally reported as a potential treatment aimed at suppressing the immune-mediated response via Fc-receptor binding, although definitive recommendations for its use are lacking.^7^

Guillain–Barré syndrome (GBS) in humans shares strong clinical and pathophysiological similarities with AIP in dogs.^2^^,^^5^ In human medicine, GBS responds favorably to therapeutic plasma exchange (TPE), leading to rapid improvement and control of disease progression.^10^^,^^11^ In dogs, the use of TPE for AIP has so far been limited to 2 published case reports, one using manual TPE^3^ and 1 membrane-based TPE,^12^ showing promising findings.

The present case series describes the clinical response of 4 dogs with AIP treated using a standardized TPE protocol.

## Case series description

**Case 1:** A 2-year-old intact male Wire Fox Terrier (9.61 kg) was presented with a 1-week history of progressive weakness. Neurological examination identified ambulatory lower motor neuron tetraparesis. Abnormalities were not detected on routine laboratory testing and infectious disease screening. Advanced diagnostics, including cerebrospinal fluid analysis and electrophysiological testing, supported a peripheral neuropathy consistent with AIP.

More detailed diagnostic findings and supportive care information are provided in Supplementary Materials S1 and S2.

Over the following 48 h, the neurological condition deteriorated to nonambulatory tetraparesis. Due to ongoing progression of the neurological deficits, the owners elected to initiate TPE. Three sessions were performed on consecutive days, processing a total of 4.5 dog plasma volumes (DPVs): 1.4 DPVs during the first and second sessions and 1.7 DPVs during the third. After the first session, the dog became ambulatory, and neurological status improved steadily over the course of treatment. The dog was discharged without further treatments 11 days after presentation, with a normal gait and only persisting reluctance to walk on stairs. No relapse was reported at the 2-month recheck.

**Case 2:** A 3-year-old intact male German Spitz (5.15 kg) was presented to the neurology service with acute, progressive weakness, and dysphonia. Neurological examination revealed normal mentation and severe ambulatory lower motor neuron tetraparesis, with reduced withdrawal reflexes in the hind limbs. Routine laboratory testing and cerebrospinal fluid analysis were unremarkable. Electrophysiological findings supported a diagnosis of peripheral neuropathy consistent with AIP. Additional information in Supplementary Materials S1 and S2.

Given the severity of neurological deficits and the strong suspicion of AIP, TPE was initiated on the day of presentation. Three sessions of TPE were performed on consecutive days. A total of 4.8 times the DPV was processed: 2.0 DPVs during the first session and 1.4 DPVs during the second and third sessions.

The withdrawal reflex returned to normal after the first session, and lower motor neuron deficits had resolved by the end of the third session. At the time of discharge, 3 days later, mild dysphonia was the only persisting neurological deficit. No relapse was reported at the 1- and 2-month rechecks.

**Case 3:** A 7-month-old intact female Shiba Inu (6.75 kg) was presented to the emergency room with a 3-day history of progressive weakness and acute dysphonia. Neurological examination showed severe lower motor neuron tetraparesis and an absent panniculus reflex. Routine laboratory testing and infectious disease screening were unremarkable. Thoracic radiographs revealed megaesophagus. Electrophysiological findings supported a diagnosis of AIP. Additional information in Supplementary Materials S1 and S2.

Due to the progression to nonambulatory tetraparesis, TPE was initiated 24 h later and performed on 3 consecutive days. A total of 4.2 times the DPV was processed: 1.4 DPVs during each session. After the first session, motor function improved to an ambulatory tetraparesis, and the dysphonia resolved. The dog was discharged 5 days after presentation without any neurological abnormalities.

The dog re-presented 10 days later with recurrent weakness and showed clinical improvement after empirical pyridostigmine (Mestinon 10-mg tablets, MEDA, Wangen-Brüttisellen, Switzerland) treatment. Antibodies against nicotinic acetylcholine postsynaptic receptors remained within normal limits, and repeat thoracic radiographs showed regression of the previously diagnosed megaesophagus. Pyridostigmine therapy was discontinued 3 months after initiation, and no relapse was reported during the 12-month follow-up period.

**Case 4:** A 5-year-old spayed female West Highland White Terrier (8.7 kg) was presented to the emergency service with a 6-day history of generalized progressive weakness and acute dysphonia. Neurological examination revealed nonambulatory lower motor neuron tetraparesis. Laboratory testing revealed mild leukocytosis and neutrophilia, with no additional abnormalities detected. Infectious disease screening was negative. Electrophysiological findings supported a peripheral neuropathy consistent with AIP. Additional information in Supplementary Materials S1 and S2.

Given the clinical presentation and the suspicion of AIP, TPE was initiated 1 day after admission. Three sessions were performed on consecutive days, processing a total of 4.6 times the DPV: 1.6 DPVs during the first session, and 1.5 DPVs during the second and third sessions. After the first session, the dog regained ambulation, and barking resumed after the second session. The dog was discharged 8 days after presentation with no neurological abnormalities. No relapse was reported at regular rechecks over the following 2 years.

## Description of TPE protocol

All TPE sessions were performed using a membrane-based system and lasted between 90 and 120 min. The DPV was estimated as 90 mL × body weight (kg) × (1 − PCV). Replacement fluid consisted of 50% Plasma-Lyte (Plasma-Lyte A, Baxter AG, Glattpark [Opfikon], Switzerland) and 50% fresh frozen plasma. Circuit anticoagulation was maintained using 30% anticoagulant citrate dextrose solution (Anticoagulant Citrate Dextrose Solution A Lösung, TS 14005, 500 mL, Fresenius Kabi AG, Kriens, Switzerland). The infusion rate was automatically adjusted by the TPE device to maintain a constant circuit citrate concentration of 3 mmol/L. Calcium supplementation with 10% calcium gluconate (Calciumgluconat B. Braun 10% Injektionslösung, B. Braun Medical AG, Sempach, Switzerland) was administered through the return line of the extracorporeal circuit to maintain normocalcemia. Standard monitoring was performed during all sessions.

In all dogs, a double-lumen hemodialysis catheter was aseptically inserted into the right jugular vein using a modified Seldinger technique. The catheter was removed 1 day after the third TPE session in all dogs.

No clinically relevant complications related to the extracorporeal therapy were observed during or between the sessions.

## Discussion

In this case series, we report the early and successful use of TPE in 4 dogs with suspected AIP. The decision to initiate TPE was based on the clinical and pathophysiological similarities between AIP and GBS in humans, for which TPE is widely recognized as a standard emergency therapy.^10^^,^^11^^,^^13^^,^^14^

Acute idiopathic polyradiculoneuritis is an immune-mediated peripheral neuropathy characterized by rapidly progressive lower motor neuron deficits.^1^^,^^3^^,^^7^ All dogs in this case series developed severe neurological deficits, including acute tetraparesis, decreased spinal reflexes, and an inability to bark, within 4-7 days before presentation.

Electrophysiological evaluation represents a cornerstone of AIP diagnosis. However, during the initial phase (typically within the first 7 days), an EMG might be normal, whereas a reduction in nerve conduction velocity is more consistently detectable.^2^

One dog relapsed with features suggestive of myasthenia gravis (MG), including fluctuating weakness and a response to pyridostigmine despite negative acetylcholine receptor antibodies.^15–17^ This might reflect early testing or seronegative MG, which has recently been documented in up to 22% of dogs with MG.^17^ In this case, discontinuation of pyridostigmine therapy was not associated with a relapse or recurrence of the clinical signs.

In AIP, supportive care—including physiotherapy, monitoring, and the prevention of complications such as aspiration pneumonia—is commonly recommended. However, recovery can take several weeks to months.^1^^,^^7^^,^^8^ Immunosuppressive corticosteroids have historically been used in dogs with suspected AIP, but their benefit remains controversial and is not consistently associated with improved clinical outcomes. In addition, concerns exist regarding potential adverse effects, including muscle weakness and delayed neuromuscular recovery, particularly in recumbent dogs.^1^^,^^7^ Immunomodulatory treatments, such as IVIg or TPE, have recently been associated with shorter recovery times in dogs.^3^^,^^7^^,^^8^^,^^12^ In veterinary medicine, the use of IVIg is reported in 10 dogs, with a median time to improvement and return to ambulation of 27.5 days after IVIg treatment.^7^ In contrast, dogs managed with supportive care alone commonly regain ambulation only after several weeks to months, with reported median recovery times often exceeding 20-30 days.^1^^,^^7^^,^^8^

None of the dogs in this case series received corticosteroids, IVIg, or other immunosuppressive treatments as part of their treatment protocol. All dogs showed measurable neurological improvement within 24 h of the first TPE session, regained ambulatory function by the completion of the initial session, and were discharged within 5-11 days of presentation. While spontaneous recovery is reported in dogs with AIP managed conservatively, the consistent temporal association between TPE initiation and neurological improvement across all 4 cases suggests a treatment effect beyond the expected natural disease course. Similar rapid clinical responses have been described in 2 recent publications in which manual or membrane-based TPE was applied in 2 dogs with AIP.^3^^,^^12^ One dog regained ambulation 48 h after the last plasmapheresis treatment.^12^ By comparison, in all dogs of the present case series, acute motor dysfunction had resolved by the completion of the 3-session protocol in all dogs. Continued hospitalization thereafter primarily reflected clinical management decisions rather than persistent neurological deterioration.

Therapeutic plasma exchange aims to remove circulating autoantibodies, immune complexes, complement components, and inflammatory mediators from the plasma. In immune-mediated polyneuropathies such as GBS, plasmapheresis could interrupt the pathological autoimmune response targeting peripheral nerves, thereby improving clinical outcomes and accelerating recovery.^18^^,^^19^ The decision to initiate extracorporeal therapy in the present cases was therefore based on the severity and rapid progression of neurological deficits, with the goal of reducing the circulating antibody and immune complex load early in the disease course.

Although antibody concentration was not measured, exchanging 1.5-2 DPVs per session is expected to result in a marked reduction of circulating antibodies.^20^ All dogs underwent 3 consecutive TPE sessions, for a total of 4.2-4.8 DPVs exchanged. This therapeutic target appeared effective in interrupting the pathophysiological course and reducing the suspected antibody load in all dogs. Similar findings are reported in a dog with AIP that underwent TPE with processing of 5.4 DPVs and experienced a near-complete reduction in antigen-specific IgG.^12^ Furthermore, no signs of clinical worsening were observed in our dogs, suggesting the absence of a rebound effect. These findings suggest that TPE is an effective therapy for dogs with AIP, with an exchange of 1.5-2 DPVs per session representing a reasonable treatment target.^21^ Additional information on circulating antibody immune complex removal by TPE is provided in Supplementary Material S3.

The favorable response observed in our dogs might be partly attributable to the early initiation of TPE. Polyradiculoneuritis can induce progressive demyelination of peripheral nerves through antibody-mediated inflammation.^1^^,^^18^ Early removal of pathogenic antibodies and immune complexes might limit ongoing demyelination and its consequences. Whether a similar benefit occurs in dogs with prolonged clinical signs and more advanced demyelination remains unknown.

In all dogs of this series, TPE was initiated within 2-8 days from the onset of neurological deficits, which likely contributed to the favorable outcomes observed. Although this supports the concept that early intervention might be beneficial, definitive criteria regarding optimal timing for initiation, treatment frequency, and total exchange volume are currently lacking. The timing of TPE initiation might represent an important determinant of clinical outcome. In human GBS, early initiation of plasma exchange, ideally within the first 7-14 days after symptom onset, is associated with faster motor recovery compared to delayed treatment.^11^^,^^14^^,^^18^ Therapeutic benefit appears to diminish when immunomodulatory therapy is initiated later in the disease course. Although comparable timing recommendations are not established in veterinary medicine, these findings support early TPE consideration in dogs with suspected AIP and rapidly progressive neurological deficits. Optimal treatment protocols remain to be defined; therefore, the proposed protocol is based on theoretical plasma exchange kinetics and empirical experience rather than established clinical evidence.

Potential complications of TPE are described in both veterinary and human medicine, including hemodynamic instability, electrolyte imbalances, and transfusion reactions.^19^ In this case series, no complications occurred during or after TPE in any of the dogs. Rapid recovery might reduce complications and hospitalization time, offering both clinical and financial benefits.

Limitations of this case series include the small sample size, the lack of a control group for direct comparison between different treatment strategies (eg, IVIg), and the unavailability of recently identified diagnostic biomarkers, such as anti-GM2 ganglioside and anti-GalNAc-GD1a IgG. These antibodies have been experimentally demonstrated in the serum of dogs with AIP,^9^ but were not commercially available at the time these dogs were treated, limiting the ability to assess their diagnostic utility or the immunological efficacy of TPE.

Other technical and physiologic factors besides exchanged plasma volume might influence TPE efficacy, including vascular access performance, antibody distribution between intra- and extravascular compartments, redistribution kinetics, and ongoing antibody production. These factors might affect the rate and extent of antibody removal and should be considered when interpreting treatment responses.^19^^,^^22^

In conclusion, TPE appeared to be associated with rapid clinical improvement and neurological recovery in 4 dogs with severe AIP, compared with timelines reported for standard supportive care or IVIg in the veterinary literature.

## Supplementary Material

Supplementary_File_aalag090

## Off-label antimicrobial declaration

The authors declare no off-label use of antimicrobials.

## Institutional animal care and use committee or other approval declaration

The authors declare no institutional animal care and use committee or other approval was needed.

## Abbreviations

AIP: acute idiopathic polyradiculoneuritis
EMG: electromyography
GBS: Guillain–Barré syndrome
IVIg: intravenous immunoglobulin
MG: myasthenia gravis
DPVs: dog plasma volumes
TPE: therapeutic plasma exchange
