# Supplementary material for: Clinical response of acute idiopathic polyradiculoneuritis treated with therapeutic plasma exchange in four dogs
Source: J Vet Intern Med. 2026 May 18;40(3):aalag090. doi: 10.1093/jvimsj/aalag090 (PMC13182567; doi:10.1093/jvimsj/aalag090)
Supplement: Supplementary_File_aalag090 [file supplementary_file_aalag090.docx]

**Supplementary File for Review:**

**Supplementary Material 1. Detailed diagnostic findings for individual cases**

**Case 1**

Hematology and serum biochemistry results were unremarkable, and the 4Dx Snap test (SNAP 4Dx Plus Test, IDEXX, Hoofddorp, The Netherlands) was negative. Antibody titers for *Toxoplasma gondii* and *Neospora caninum* (Toxoplasma IgG-Ab Positive Plasma, SeraCare, Milford, USA; *Neospora caninum*, IFAT IgG-Ab, Sigma-Aldrich, Merck, St. Louis, USA) were unremarkable. Thoracic radiographs, cerebrospinal fluid analysis, and electromyography (EMG) (Keypoint G4 EMG/NCS/EP Workstation, Alpine Biomed ApS, Skovlunde, Denmark) were unremarkable. Nerve conduction velocity of the tibial nerve was reduced predominantly in the distal segment (48 m/s), and the waveform was mildly polyphasic.

**Case 2**

Hematology and serum biochemistry results were unremarkable, and cerebrospinal fluid analysis was unremarkable. Electromyography revealed spontaneous fibrillation potentials and positive sharp waves in the hind limb muscles. Nerve conduction velocity of the tibial nerve was reduced to 40 m/s in the distal segment.

**Case 3**

Hematology and serum biochemistry results were unremarkable. Antibodies against nicotinic acetylcholine receptors (ACh-receptor Ab reflex panel, UC San Diego Health, San Diego, USA), as well as *Toxoplasma gondii* and *Neospora caninum*, were unremarkable. Megaesophagus was identified on thoracic radiographs. Cerebrospinal fluid analysis was unremarkable, and EMG revealed spontaneous fibrillation potentials and positive sharp waves in all limbs. Nerve conduction velocity of the tibial nerve was reduced to 20 m/s.

**Case 4**

Hematology and serum biochemistry showed mild leukocytosis and neutrophilia but were otherwise unremarkable. Antibodies against nicotinic acetylcholine receptors, tick-borne meningoencephalitis (FSME, Virus-AK IgM/IgG, ALOMED Müller GmbH & Co., Radolfzell am Bodensee, Germany), *Neospora caninum*, and *Toxoplasma gondii* were unremarkable. Thoracic radiographs were unremarkable. Electromyography revealed pathological spontaneous activity in distal muscles, including the plantar and palmar interossei, tibialis cranialis, and extensor carpi radialis longus muscles. Nerve conduction velocity of the tibial nerve was reduced to 40 m/s.

**Supplementary Material 2. Detailed case descriptions and supportive care**

This supplementary material provides detailed descriptions of supportive care and adjunctive treatments administered in each case.

**Case 1**The dog was hospitalized for monitoring and supportive care, including intravenous crystalloid fluids adjusted to hydration status, physiotherapy, bladder management, and antiemetic and analgesic therapy as clinically indicated.

**Case 2**

Supportive care was provided concurrently and included intravenous crystalloids, physiotherapy, antiemetic and analgesic therapy as clinically indicated, and intravenous antibiotics for a catheter-site infection.

**Case 3**

The dog was hospitalized for monitoring and supportive care, including intravenous fluids, physiotherapy, bladder management, and antiemetic and analgesic therapy as clinically indicated.

**Case 4**

The dog was hospitalized for monitoring and supportive care, including intravenous crystalloids, physiotherapy, bladder management, antihistamines for mild facial swelling during plasma administration, and antiemetic and analgesic therapy as clinically indicated.

**Supplementary Material 3. Detailed information on circulating antibody immuncomplexes removal by TPE**

The treatment protocol used in this series, processing 4.2–4.8 PPVs over three consecutive sessions, represents a practical and effective scheme that may serve as a reference for future cases. The removal efficiency of plasma solutes during TPE is directly related to the number of processed PPVs. Exchanging two PPVs results in approximately an 87% reduction of the solutes present at the start of the procedure, whereas exchanging 1.5 PPVs yields about a 78% reduction. This relationship is described by the equation: Y = e⁻ˣ, where Y is the proportion of immunoglobulins remaining at the end of treatment, e is the base of the natural logarithm, and x represents the number of PPVs exchanged during the session (21).
